# Identifying nucleic acid-associated proteins in *Mycobacterium smegmatis* by mass spectrometry-based proteomics

**DOI:** 10.1186/s12860-020-00261-6

**Published:** 2020-03-23

**Authors:** Nastassja L. Kriel, Tiaan Heunis, Samantha L. Sampson, Nico C. Gey van Pittius, Monique J. Williams, Robin M. Warren

**Affiliations:** 1 DST-NRF Centre of Excellence for Biomedical Tuberculosis Research; South African Medical Research Council Centre for Tuberculosis Research; Division of Molecular Biology and Human Genetics, Faculty of Medicine and Health Sciences, Stellenbosch University, PO Box 19063, Tygerberg, Cape Town, 7505 South Africa; 2 Institute for Cell and Molecular Biosciences, Newcastle University, Newcastle upon Tyne, UK; 3 Present address: Department of Molecular and Cell Biology, University of Cape Town, Cape Town, South Africa

**Keywords:** RNA polymerase, Mycobacterium, Affinity-purification, Nucleic acid-associated proteins

## Abstract

**Background:**

Transcriptional responses required to maintain cellular homeostasis or to adapt to environmental stress, is in part mediated by several nucleic-acid associated proteins. In this study, we sought to establish an affinity purification-mass spectrometry (AP-MS) approach that would enable the collective identification of nucleic acid-associated proteins in mycobacteria. We hypothesized that targeting the RNA polymerase complex through affinity purification would allow for the identification of RNA- and DNA-associated proteins that not only maintain the bacterial chromosome but also enable transcription and translation.

**Results:**

AP-MS analysis of the RNA polymerase β-subunit cross-linked to nucleic acids identified 275 putative nucleic acid-associated proteins in the model organism *Mycobacterium smegmatis* under standard culturing conditions. The AP-MS approach successfully identified proteins that are known to make up the RNA polymerase complex, as well as several other known RNA polymerase complex-associated proteins such as a DNA polymerase, sigma factors, transcriptional regulators, and helicases. Gene ontology enrichment analysis of the identified proteins revealed that this approach selected for proteins with GO terms associated with nucleic acids and cellular metabolism. Importantly, we identified several proteins of unknown function not previously known to be associated with nucleic acids. Validation of several candidate nucleic acid-associated proteins demonstrated for the first time DNA association of ectopically expressed MSMEG_1060, MSMEG_2695 and MSMEG_4306 through affinity purification.

**Conclusions:**

Effective identification of nucleic acid-associated proteins, which make up the RNA polymerase complex as well as other DNA- and RNA-associated proteins, was facilitated by affinity purification of the RNA polymerase β-subunit in *M. smegmatis*. The successful identification of several transcriptional regulators suggest that our approach could be sensitive enough to investigate the nucleic acid-associated proteins that maintain cellular functions and mediate transcriptional and translational change in response to environmental stress.

## Background

Nucleic acid-associated proteins are required to regulate and execute the transcriptional responses required to sustain cellular homeostasis or to adapt to environmental stresses experienced. DNA-associated proteins are known to include DNA polymerases, transcription factors, nucleases and nucleoid-associated proteins (bacteria) which aid in transcriptional regulation, DNA repair, recombination and stabilization of the bacterial nucleoid [1–3]. Likewise, RNA-associated proteins which include the RNA polymerase complex, ribosomal proteins, ligases and helicases have been shown to influence RNA stability, transport, localisation and translation [4, 5]. The identification and investigation of DNA- and RNA-associated proteins are known to be problematic as some protein populations, such as transcriptional regulators, are often low in abundance or exhibit weak DNA binding abilities [6, 7]. High-throughput methodologies used in the past to identify these proteins included sucrose density centrifugation followed by mass spectrometry, which was effective in the identification of nucleic acid-associated proteins, but these workflows are prone to protein contaminants from other cellular fractions [8]. Other approaches have made use of nonspecific, specific, single- or double-stranded DNA columns to affinity purify DNA-binding proteins for identification [9]. More recently, an improved affinity purification-mass spectrometry (AP-MS) approach was developed to aid in the identification of unknown DNA-binding proteins to known DNA sequences and another approach, Epi-Decoder, made use of Tag-chromatin immunoprecipitation-Barcode-Sequencing to identify DNA-binding proteins associated with known DNA loci through DNA sequencing [10, 11]. Advances in RNA proteomics has seen methodologies such as enhanced RNA interactome capture (eRIC) and orthogonal organic phase separation (OOPS) efficiently identify RNA-binding proteins [12, 13].

The identification of these nucleic-acid associated proteins has aided in our understanding of the proteins which are required to sustain cellular homeostasis or adapt to environmental stress. In mycobacteria, high-throughput technologies such as microarrays, ChIP-seq and RNA-seq have been instrumental in understanding the transcriptional responses necessary for bacterial survival and cell homeostasis [1, 14–18]. One limitation of ChIP-seq and microarrays are that these methodologies investigate transcriptional regulators individually, which can be problematic when adaptation to adverse environmental conditions involves multiple transcriptional regulators and regulatory elements acting in concert. Understanding adaptation to environmental stress is further complicated by the concurrent regulation of genes through transcriptional regulators, as seen in *M. tuberculosis* with DevR and Lsr2 which are both induced by hypoxia and redox stress [19–23]. The overlap in gene regulation by some transcriptional regulators may suggest that several regulatory elements known to be associated with specific environmental cues may have unknown functions. A new approach that would aid in the identification of regulatory proteins required by bacteria for cellular homeostasis or to adapt to environmental stress conditions is therefore needed. The development of a global, high-throughput approach, which can be used to identify and characterise these regulatory proteins, will allow us to better understand complex transcriptional cascades.

In this study, we aimed to identify mycobacterial nucleic acid-associated proteins required for maintaining cell homeostasis under standard laboratory conditions by targeting the RNA polymerase (RNAP) complex as a “tag” for nucleic acids in the non-pathogenic model organism *Mycobacterium smegmatis*. We successfully applied an affinity purification-mass spectrometry (AP-MS) approach, to identify not only proteins that make up the RNAP complex but also other proteins that are known to be associated with nucleic acids and the RNAP complex. These include 12 uncharacterised proteins with no known predicted cellular functions or known association with nucleic acids. To validate the ability of our AP-MS approach to identify nucleic acid-associated proteins, we sought to demonstrate DNA association for several identified proteins. We propose that our approach can be used to investigate protein populations required by mycobacterial species to sustain cellular stability during normal growth or under stress, and that this approach may have utility in other bacterial species.

## Results

### Identifying nucleic acid-associated proteins in *M. smegmatis*

**Fig. 1:**
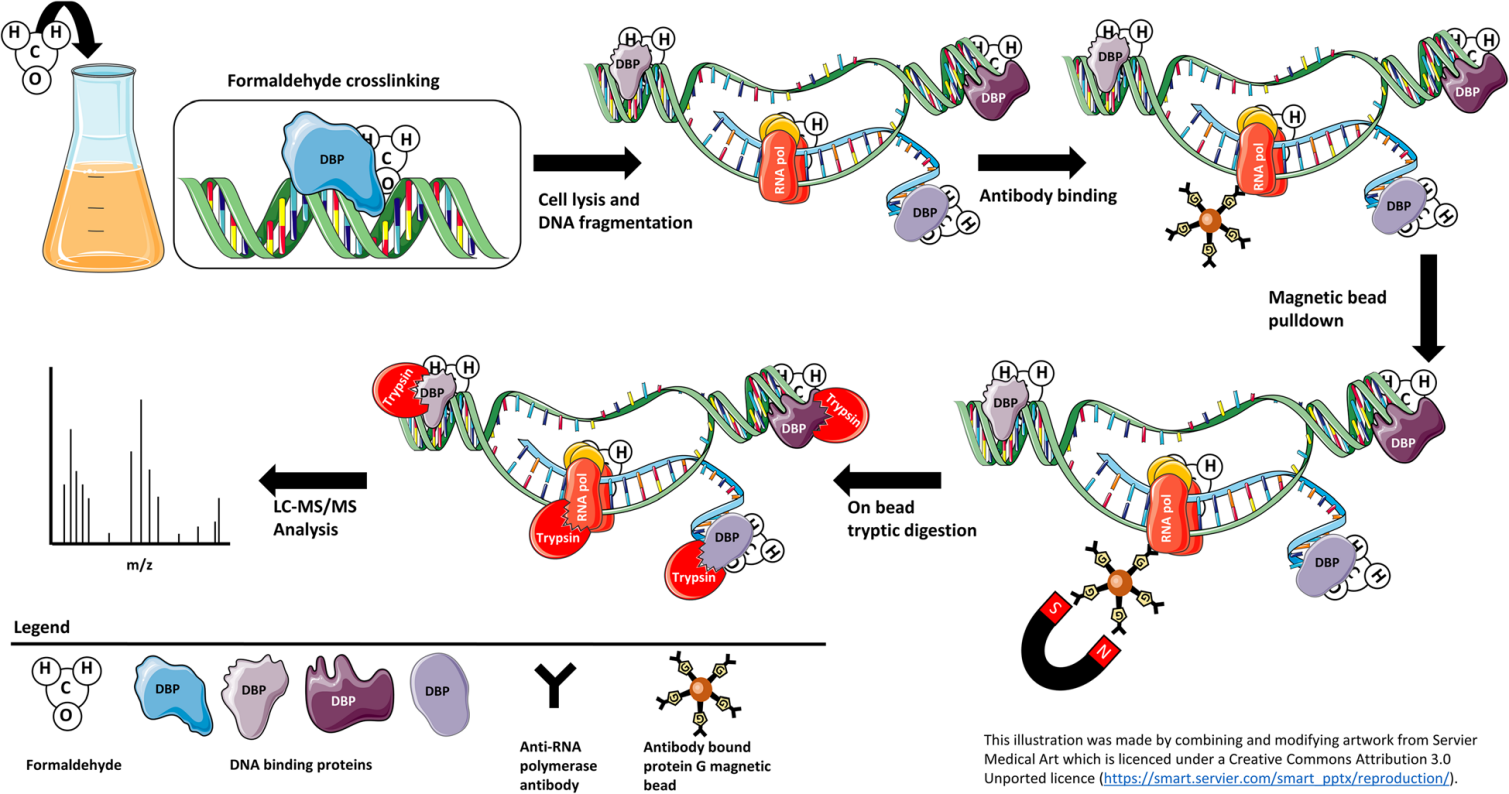
Isolation of nucleic acid-associated proteins. Formaldehyde was introduced into bacterial cultures to stabilize protein-nucleic acid interactions through crosslinking. Bacterial cells were lysed and protein-DNA complexes fragmented prior to immunoprecipitation using an anti-RNA polymerase antibody immobilized on protein G coated magnetic beads. Immunoprecipitated proteins were digested of magnetic beads using trypsin and mass spectrometry analysis was performed to identify nucleic acid-associated proteins

To identify possible RNAP and nucleic acid-associated proteins, we affinity purified the RNAP complex from formaldehyde treated *M. smegmatis* cell lysates using an anti-RNAP β-subunit antibody immobilized on protein G magnetic beads (Fig. 1). Formaldehyde is a four-atom molecule that chemically crosslinks protein-nucleic acid or protein-protein complexes that are ~ 2 Å apart, allowing for the successful isolation of interacting proteins but also enabling the isolation of any closely associated proteins [24, 25]. We predicted that formaldehyde treatment of *M. smegmatis* cultures would not only result in the stable isolation of the RNAP complex and its associated proteins, but also enrich for proteins associated with DNA and RNA molecules. To control for non-specific interactions during immunoprecipitations we included a protein G Dynabead control as well as a non-specific antibody control, protein G Dynabeads coated with anti-human heavy chain seven myosin antibody.

**Fig. 2:**
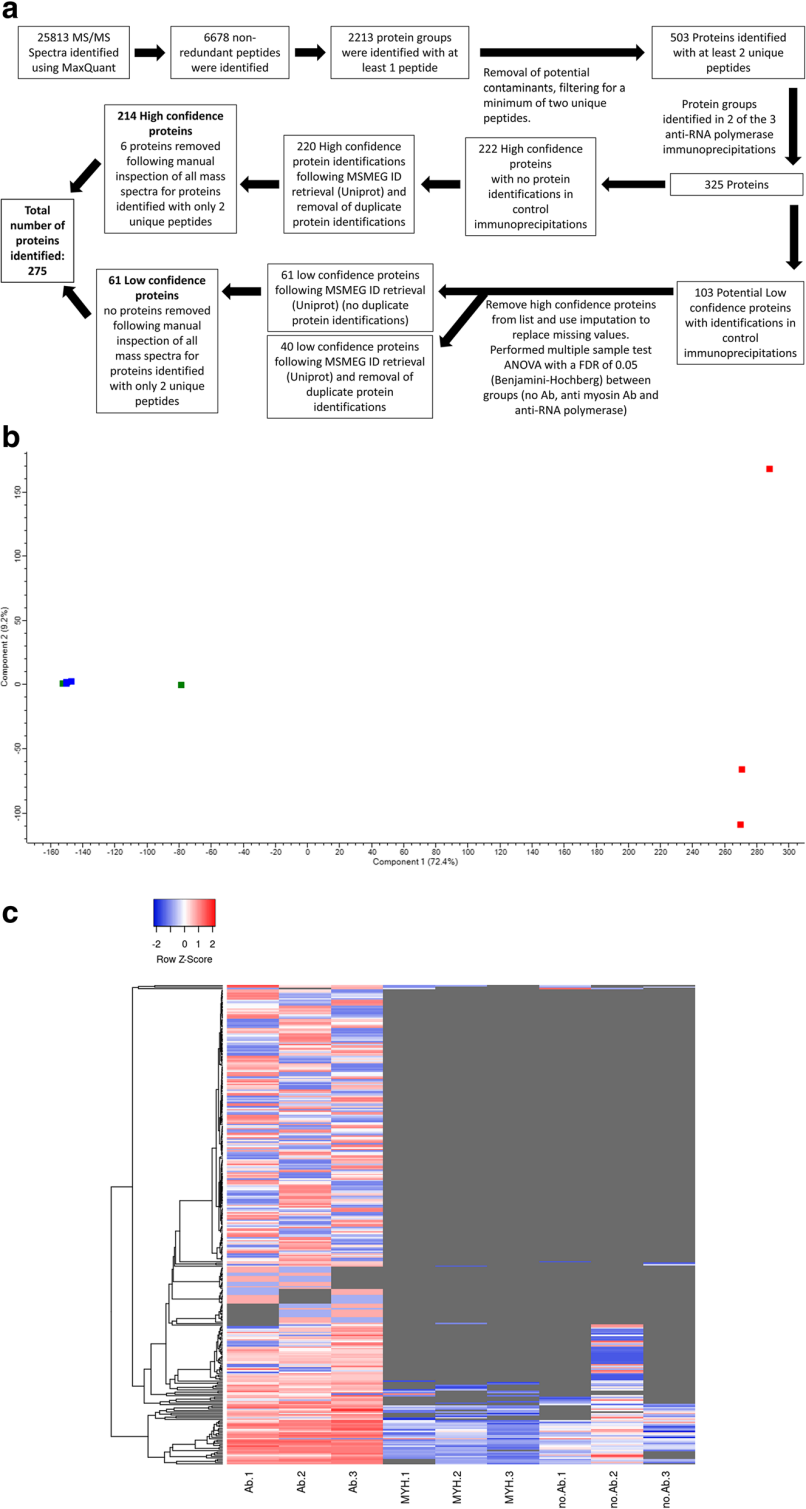
Identification of nucleic acid-associated proteins from mass spectrometry data. **a**. Diagram demonstrating data analysis of mass spectrometry data. Three hundred and twenty-five protein groups were identified in at least two of the three immunoprecipitations, 222 of these were identified as high confidence protein groups and were not identified in any of the control immunoprecipitations. Multiple sample testing with an FDR of 0.05 and a fold change of two was used to identify 63 low confidence proteins from 103 possible low confidence proteins. Uniprot protein annotations were mapped to MSMEG database annotations, removing Uniport annotations that matched to MSMEI or LJ100 database annotations (assigned during automated database searching), resulting in the identification of 220 high confidence and 61 low confidence proteins. Following the manual inspection of spectra for all proteins identified with a minimum of 2 unique peptides, a total of 275 proteins were identified, of which 214 were high confidence proteins and 61 were low confidence proteins. **b**. Principal component analysis revealed separate clustering of replicate anti-RNAP immunoprecipitations and control immunoprecipitations for the 503 protein groups with two unique peptides. Anti-RNAP immunoprecipitations are displayed in red blocks on the right with protein G Dynabead control immunoprecipitations displayed in green and anti-MYH7 control immunoprecipitations displayed in blue on the left. **c**. The heatmap shows the clustering of the anti-RNA polymerase immunoprecipitation on the left and control immunoprecipitations right and center. The red colour is indicative of a higher abundance and the blue of a lower abundance of a protein within an immunoprecipitated sample. The grey colouring within the heatmap is representative of the absence of a protein within that immunoprecipitation

**Fig. 3:**
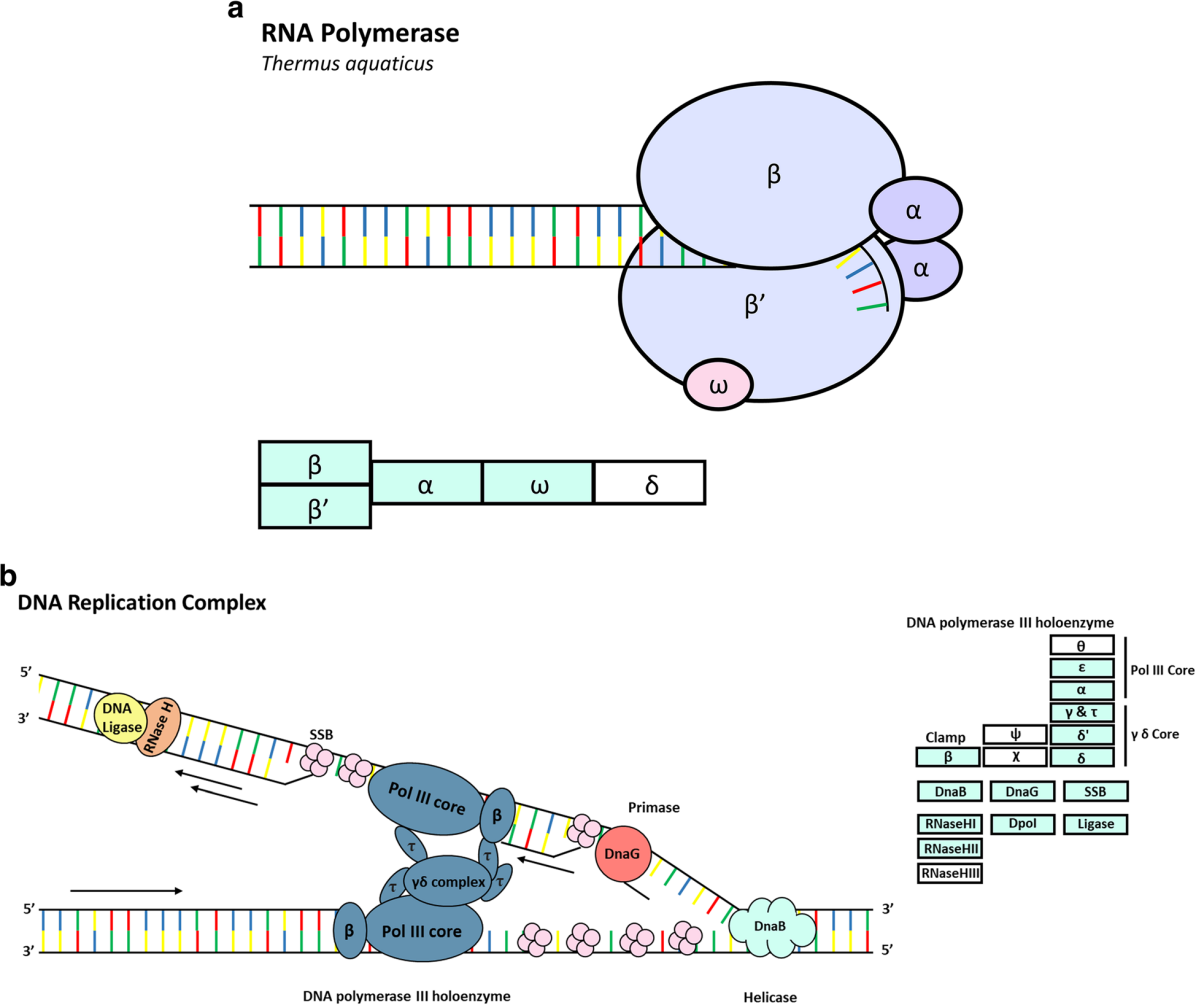
Identification of transcription and translation machinery. **a**. The figure displays the RNA polymerase structure and identified subunits. *M. smegmatis* identified RNA polymerase complex subunits were mapped using the KEGG pathway mapping tool and are indicated in green in the blocks. **b**. The figure displays the DNA replication complex and identified subunits. *M. smegmatis* identified DNA replication complex subunits were mapped using the KEGG pathway mapping tool and are displayed in green in the blocks

Using this approach, we identified 6678 unique peptides that mapped to 503 protein groups with a least two unique peptides (Fig. 2a). Principal component analysis revealed separate clustering of replicate anti-RNAP and control immunoprecipitations before filtering for protein detection in at least two of three anti-RNAP immunoprecipitations (Fig. 2b). These plots demonstrate that clustering of anti-RNAP immunoprecipitations was not a result of filtering for proteins detected in at least two of the three anti-RNAP immunoprecipitations. Hierarchical clustering using a heatmap demonstrated separate clustering of anti-RNAP and control immunoprecipitations for the 325 proteins that were identified in least two of the three anti-RNAP immunoprecipitations and demonstrated the ability of our AP-MS approach to limit contaminant protein identifications within control immunoprecipitations (Fig. 2c). We identified 214 high confidence proteins, which were identified within at least two of the three anti-RNAP immunoprecipitations, but not in any of the control immunoprecipitations (Additional file 2: Table S2-S4). We further applied a set of less stringent criteria to identify a set of lower confidence proteins (Additional file 2: Table S5). These were defined as proteins also identified in the negative control immunoprecipitations, but detected in higher abundance in the anti-RNAP β-subunit immunoprecipitations than in the control immunoprecipitations. Label-free quantification (LFQ) data was used to identify the low confidence nucleic acid-associated proteins by performing a multiple sample test ANOVA with an FDR of 0.05 using the Benjamini-Hochberg correction. This identified 61 low confidence proteins (Fig. 2a, Additional file 2: Table S2-S5). Of the 275 proteins identified (214 high confidence and 61 low confidence proteins), we identified the core proteins which are known to make up the RNAP complex in bacterial organisms (Fig. 3a). Specific *M. smegmatis* RNAP complex proteins identified included RpoA, −B, −C, −D (Sig A)*, −Z* as well as two other sigma factors, SigH* and MysB* (Additional file 2: Table S2) (* denotes high confidence proteins). Our approach also successfully identified proteins that are known to make up the DNA replication complex in bacteria (Fig. 3b). These protein identifications confirmed the validity of our approach to identify nucleic acid-associated proteins.

We next sought to determine which functional attributes were enriched within our list of protein identifications, to do this we performed a GO enrichment analysis using the Gene Ontology Enrichment Analysis Software Toolkit (GOEAST) [26]. Classification of enriched GO terms using Reduce Visualize Gene Ontology (REVIGO) revealed that 104 non-redundant enriched GO terms were associated with the functional category biological processes (Additional file 2: Table S6), 60 with molecular function (Additional file 2: Table S7) and 23 with cellular processes (Additional file 2: Table S8) [27]. Hierarchical clustering of the GO annotations in the functional categories biological processes (Additional file 1: Figure S1) and molecular function (Additional file 1: Figure S2) revealed an enrichment for several GO terms associated with nucleic acids. Enriched GO terms within these categories included “DNA replication”, “translation”, “DNA metabolic process”, “nucleic acid binding” and “RNA binding”. Unsurprisingly, the GO annotation “ribosome” was the most enriched term in the functional category cellular components (Additional file 1: Figure S3).

**Fig. 4:**
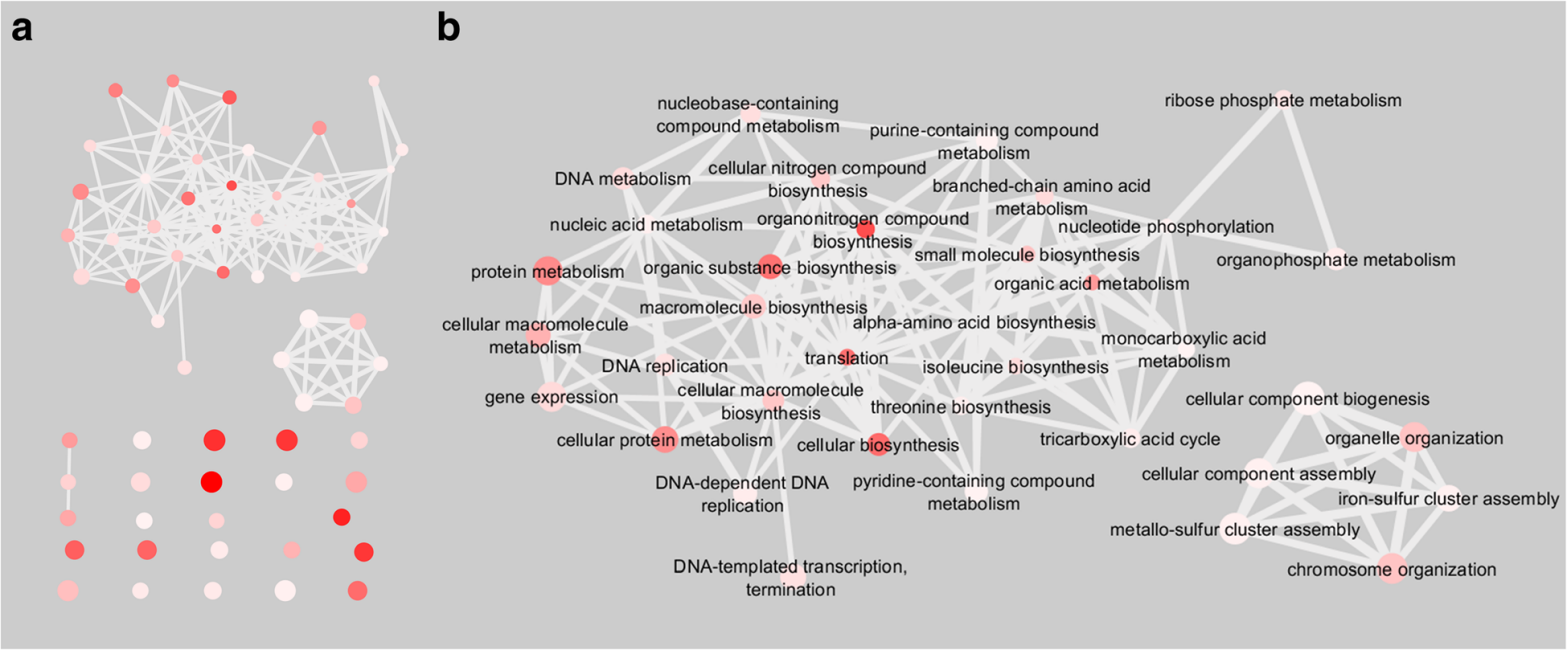
Enrichment of GO terms associated with nucleic acids for biological processes. Each GO terms is represented as a node, with interactions between nodes representing the similarities of GO terms. The size of each node is representative of the frequency of each GO term within our dataset. The colour of each node is indicative of the –log10 *p*-value, with a darker colour representing a more significant enrichment. **a**. All enriched biological processes GO terms. **b**. A network of similar GO terms was isolated from A. using identifiers such as DNA, chromosome, transcription, gene, RNA, ribosome, translation, nucleotide as well as all first connecting neighbours

**Fig. 5:**
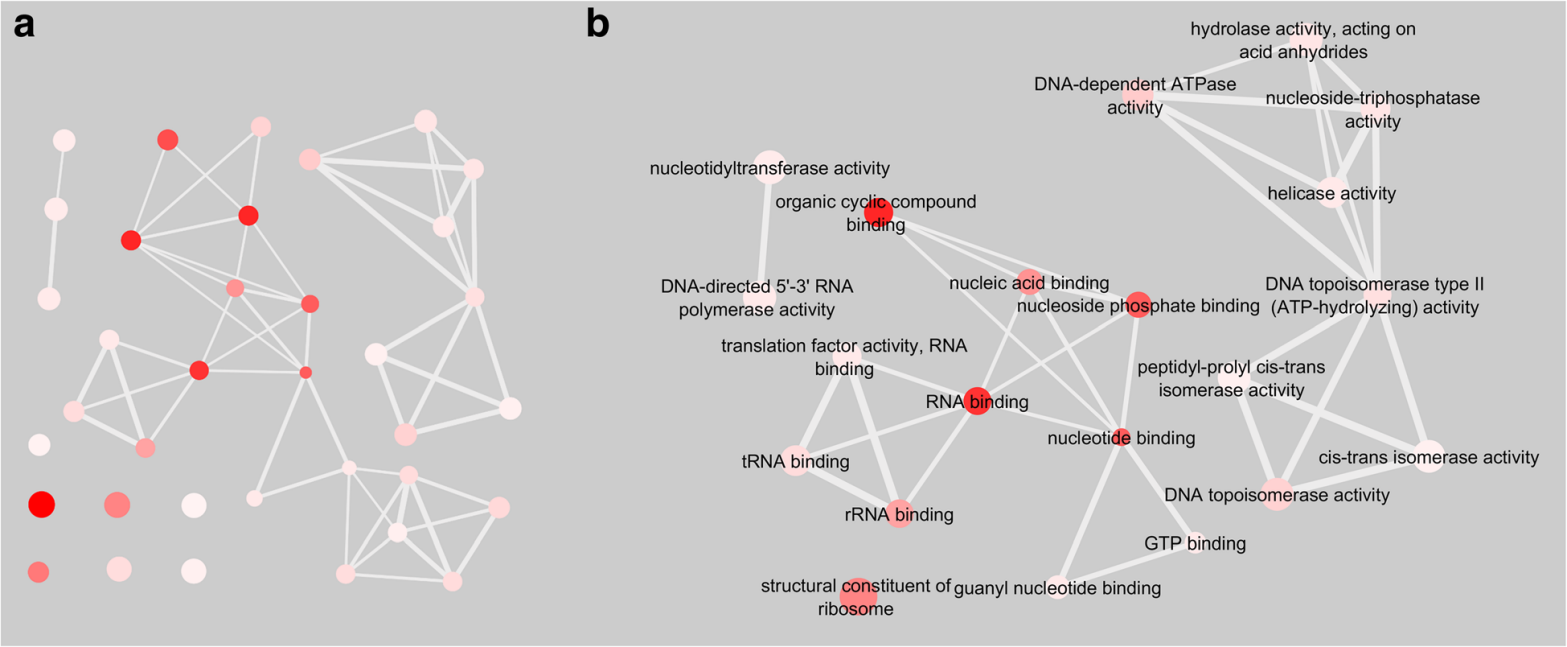
Enrichment of GO terms associated with nucleic acids for molecular function. GO terms are represented as nodes, with interactions connecting similar GO terms. The size of each node is indicative of its frequency within our dataset and the colour represents the –log10 p-value. **a**. Enriched GO molecular function GO terms. **b**. A network of similar GO identities was generated using the identifiers DNA, chromosome, transcription, gene, RNA, ribosome, translation, nucleotide and first connecting neighbours

To better demonstrate the relationships between enriched GO terms in the functional categories biological processes and molecular functions, we made use of functional annotation network graphs generated using REVIGO and Cytoscape (Figs. 4a and 5a) [27, 28]. These functional annotation networks displayed enriched GO terms as nodes, of which the colour is indicative of –log10 *p*-value of the GO term enrichment. Highly similar GO terms were connected by edges. Functional annotation network graphs for biological processes (Fig. 4a) and molecular function (Fig. 5a) demonstrated that several GO terms associated were enriched and connected, suggesting that the proteins identified in this study may have similar or related functional attributes. As we expected, our approach also facilitated the enrichment of proteins in close proximity or in direct contact with the RNAP complex, as can be seen with the enriched and connected GO terms related to nucleic acids (Figs. 4b and 5b). Metabolic pathway mapping using the Kyoto Encyclopedia of Genes and Genomes revealed that proteins identified in this study are not only predicted to be required for nucleic acid metabolism, but also for energy, lipid, carbohydrate and amino acid metabolism (Additional file 1: Figure S4) [29].

### DNA association of identified proteins

Annotation of the *M. smegmatis* genome revealed that it encodes for approximately 6938 proteins [30]. Gene ontologies have been used to suggest functional attributes for all predicted proteins based on the homology of conserved domains, however, very little of this has been corroborated using functional studies. A subset of genes, 24.6% (1708 genes) are believed to encode for hypothetical proteins, of which 1040 genes are thought to encode for conserved hypothetical proteins [30]. Given the high proportion of predicted hypothetical proteins that may be encoded by *M. smegmatis* and the high number of proteins that still remains to be functionally investigated, several DNA- and RNA-associated proteins may still remain unidentified.

**Fig. 6:**
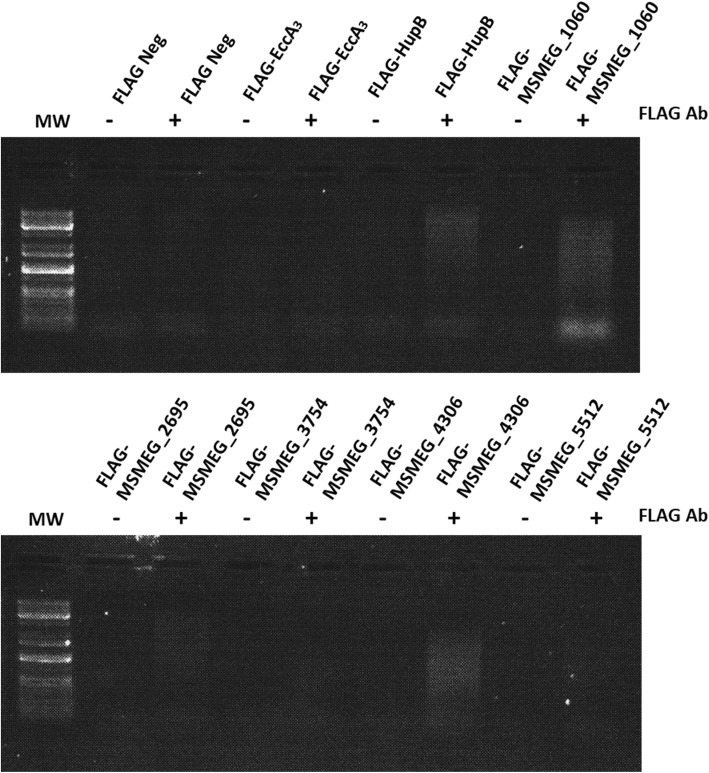
Immunoprecipitation of N-terminal FLAG-tagged *M. smegmatis* proteins. FLAG-tagged MSMEG_1060, MSMEG_2695, MSMEG_3754, MSMEG_4306 and MSMEG_5512 were episomally expressed in *M. smegmatis*. The first lane of each gel contains a GeneRuler™ 1 kb plus DNA molecular weight marker (MW), − and + indicates the absence or presence of the anti-FLAG-tag antibody. Negative controls, FLAG only and FLAG-EccA3, did not demonstrate any association with DNA following immunoprecipitation. The positive control FLAG-HupB, FLAG-MSMEG_1060, FLAG-MSMEG_2695 and FLAG-MSMEG_4306 did demonstrate DNA association following immunoprecipitation

To validate the ability of our AP-MS approach to identify nucleic acid-associated proteins, we determined whether select proteins from our high confidence list are DNA-associated. We expressed five *M. smegmatis* genes from an episomal plasmid as N-terminal FLAG-tagged proteins (Table 1, Additional file 1: Figure S5). MSMEG_1060, MSMEG_2695, MSMEG_3754, MSMEG_4306 and MSMEG_5512 are proteins of unknown function, which were selected through the identification of conserved protein domains that have been shown to be associated with nucleic acids (Table 1). Three of these proteins have *M. tuberculosis* orthologues, namely MSMEG_2695 (Rv2744c), MSMEG_3754 (Rv1691) and MSMEG_5512 (Rv0958). *M. smegmatis* cultures were treated with formaldehyde to stabilize possible DNA-protein interactions, prior to affinity purification of FLAG-tagged proteins. The nucleoid-associated protein HupB (MSMEG_2389) was selected as a positive control and the cytoplasmic component of the ESX-3 secretion system, EccA_3_ (MSMEG_0615), was selected as a negative control (Fig. 6). A protein G Dynabead control, as well as a *M. smegmatis* strain expressing the FLAG-tag alone were also included as negative controls (Fig. 6). We successfully recovered DNA from affinity purified FLAG-tagged MSMEG_1060, MSMEG_2695 and MSMEG_4306 (Fig. 6). Although this does not directly confirm DNA binding by the episomally expressed proteins, formaldehyde crosslinking does suggest that these proteins may at least be in close proximity to DNA [34]. No DNA association was found following affinity purification of FLAG-tagged MSMEG_3754 and MSMEG_5512 (Fig. 6).

**Table 1:** AP-MS identified proteins selected for validation

MSMEG Gene Annotation	*M. tuberculosis* Orthologue annotation^a^	Protein Names^b^	Protein Domain^b,c,d^	Description^c,d^
MSMEG_1060	–	Putative Lsr2 protein (Uncharacterized protein)	–	Lsr2 is a DNA-bridging protein in *Mycobacterium*.
MSMEG_2695	Rv2744c	35 kDa protein	PspA/IM30	PspA suppresses sigma54-dependent transcription, negative regulator of *E. coli* phage shock operon.
MSMEG_3754	Rv1691	Tetratricopeptide repeat (TPR)-repeat-containing protein	TPR	TPRs have shown involvement in cell cycle regulation, transcriptional control, and protein folding.
MSMEG_4306	–	Uncharacterized protein	C4-type zinc ribbon	Structural modelling suggests that Zn-ribbon domain may bind nucleic acids.
MSMEG_5512	Rv0958	Magnesium Chelatase	RNA polymerase sigma factor 54 interaction domain	Interaction with sigma-54 factor and has ATPase activity. Half of the proteins identified with this domain might belong to signal transduction two-component systems.

^a^ Obtained from Mycobrowser (https://mycobrowser.epfl.ch/), ^b^ Obtained from Uniprot (https://www.uniprot.org/), ^c^ Obtained from InterPro (https://www.ebi.ac.uk/interpro/), ^d^ Obtained from Pfam (https://pfam.xfam.org/). PspA/IM30 domain first identified in the PspA protein in *Escherichia coli* [31], Tetratricopeptide repeat (TPR) have been identified in a wide variety of proteins including transcription factors [32], structural modelling suggests nucleic acid binding by C4-type zinc ribbon [33]

## Discussion

In this study, we sought to identify nucleic acid-associated proteins in the model organism *Mycobacterium smegmatis* through the targeted purification of the RNAP complex. Our AP-MS approach successfully identified 275 proteins, of which 214 were deemed high confidence proteins (these proteins were not detected in any control immunoprecipitations) and a further 61 as low confidence proteins (proteins that were identified in a greater abundance within anti-RNAP immunoprecipitations (Fig. 2a, Additional file 2: Table S2).

Formaldehyde treatment of *M. smegmatis* cells created stable cross-links between protein-nucleic acids and protein-protein interaction complexes, thereby allowing us to investigate these complexes under near-physiological conditions [34]. Although chemical cross-linking with formaldehyde does limit our ability to distinguish between protein-nucleic acid and protein-protein interactions, in this study formaldehyde stabilization of protein-protein complexes allowed the purification of the RNAP complex through the targeting of the RNAP β-subunit. Furthermore, the introduction of possible variable modifications though formaldehyde cross-linking limits our ability to identify all immunoprecipitated proteins as only the most frequently occurring modifications were selected for automated database searching (Additional file 2: Table S1, Table S3). New methodologies such as eRIC and OOPS have made use of UV cross-linking to stabilize protein-RNA and protein-DNA interactions through the generation of “zero length” protein-nucleic acid cross-links which has successfully been used to improve ChIP-seq specificity and to investigate RNA binding proteins [12, 13, 35, 36]. However, this approach is not exempt from to the generation of protein-protein cross-links, as demonstrated by the generation of covalent cross-links between aromatic amino acids, thereby not ruling out the possibility of identifying associated proteins [37]. Inspection of the proteins identified in our study revealed that our approach successfully identified several nucleic acid associated proteins including, DNA polymerases, topoisomerases, helicases, transcription factors and ribosomes (Additional file 2: Table S2). We identified 20 uncharacterized proteins (Additional file 2: Table S2), of which 12 (MSMEG_0067, MSMEG_0243, MSMEG_0754, MSMEG_0824, MSMEG_0948, MSMEG_1165, MSMEG_1342, MSMEG_1680, MSMEG_2782, MSMEG_3020, MSMEG_3595, MSMEG_4306) had no identifying GO terms and are not known to be associated with nucleic acids or nucleic acid-associated proteins. Gene ontology enrichment analysis demonstrated that several GO terms associated with nucleic acids were enriched, suggesting an enrichment for nucleic acid-associated proteins (Additional file 2: Table S6-S7). Furthermore, our data indicates that proteins identified in this study have similar or related functions (Figs. 4 and 5). Notably, gene ontology enrichment for the functional category cellular components demonstrated an enrichment for the GO term “ribosome”, but no enrichment for any cell wall or membrane components was found. This was expected as our approach failed to identify cell wall associated proteins, apart from the cell wall synthesis proteins CwsA and Wag31. These proteins may be involved in septal and polar peptidoglycan synthesis and in the coordination of FtsZ-ring assembly in mycobacteria, suggesting that these proteins were likely identified because of their proximity to nucleic acids and nucleic acid-associated proteins [38]. Metabolic pathway mapping of identified proteins demonstrated that apart from identifying proteins required for transcription and translation, that our AP-MS approach identified proteins involved in energy, amino acid and lipid metabolism (Additional file 1: Figure S4). These “contaminant” proteins may have been cross-linked to the RNAP complex or other nucleic acid interacting proteins and included proteins known to be associated with iron-sulphur cluster assembly, energy metabolism and amino acid metabolism (Additional file 2: Table S2). The identification of these “contaminant” proteins are not unexpected as they are likely involved in the maintenance and function of the RNA and DNA polymerase complexes. Iron-sulphur clusters are important elements of several proteins, including DNA polymerases, nucleases and helicases, which are crucial enzymes for DNA replication and repair [39]. The identification of iron-sulphur cluster assembly proteins together with nucleic acid-associated proteins suggest a role for these proteins in transcriptional maintenance and execution. Likewise, energy metabolism proteins will be required for transcriptional and translational maintenance and execution.

Similar to ChIP-seq workflows, cross-linked protein-nucleic acid complexes were purified using an antibody immobilized on a solid matrix (Fig. 1). We considered the RNAP complex as a suitable “tag” for nucleic acids in the cell due to the dispersed presence of the RNAP complex throughout the *M. tuberculosis* and *M. smegmatis* genomes [18, 40]. By targeting the natively expressed RNAP complex and not an ectopically expressed tagged DNA or RNA interacting protein, we limited altering the physiological state of the organism, which could result in the identification of non-specific proteins [41]. Furthermore, mouse IgG antibodies have previously been shown to be resistant to proteolytic cleavage by trypsin under native conditions [42]. We therefore opted to elute immunoprecipitated proteins through on-bead tryptic digestion under non-denaturing conditions, to limit contamination of the immunoprecipitation by the anti-RNAP β-subunit antibody used to target the RNAP complex.

To validate the ability of our approach to identify nucleic acid-associated proteins, we demonstrated DNA association for N-terminally FLAG-tagged MSMEG_1060, MSMEG_2695 and MSMEG_4306. These results are not indicative of direct DNA binding by these proteins, since association with DNA may be as a result of cross-linking to other DNA-associated proteins. Possible DNA interaction and DNA binding sequences of these proteins remains to be investigated using approaches such as DNA foot printing, ChIP-seq or microscale thermophoresis. No DNA association by some of the other proteins investigated (MSMEG_3754 and MSMEG_5512) does not negate the possibility of DNA interaction, as the amount of DNA bound by these proteins may be too little to visualize on an agarose gel or these proteins may simply be RNA associated. These results highlight the ability of our approach to identify proteins associated with DNA and suggests that uncharacterised proteins identified in this study could be investigated as proteins likely to be involved in transcription or translation due to their proximity to the RNA polymerase complex.

## Conclusions

In this study, we successfully identified proteins associated with the RNAP complex under standard laboratory growth conditions in *M. smegmatis.* We propose that our AP-MS approach can successfully be applied to study the regulation of adaptation to stress in mycobacterial species, and be adapted for use in other bacteria.

## Methods

### Bacterial strains and culture conditions

*Escherichia coli* XL-1 blue (Stratagene) was used to propagate plasmid DNA constructs. *E. coli* was cultured in Luria-Bertani liquid broth (LB) or on solid LB agar plates at 37 °C, supplemented with antibiotics as required at the following concentrations: kanamycin 50 μg/mL and hygromycin 150 μg/mL. All mycobacterial work was performed using the laboratory strain *M. smegmatis* mc^2^155 grown in Difco™ Middlebrook 7H9 Albumin-Dextrose (AD) and 0.05% Tween-80 at 37 °C with shaking or on BBL™ Seven H11 Agar AD Base plates at 37 °C for 2–3 days [43]. Culture media was supplemented with antibiotics kanamycin (25 μg/mL) and/or hygromycin (50 μg/mL) as appropriate.

### Chemicals, antibodies and oligonucleotides used in this study

All chemicals used in this study were purchased from either Sigma-Aldrich or Merck South Africa, unless otherwise stated. Monoclonal antibodies to the beta subunit of RNA polymerase from *E. coli* (clone 8RB13) and human myosin heavy chain 7 (MYH7) (clone sc-53,089) were purchased from Santa-Cruz, United States of America (USA). A mouse derived monoclonal anti-FLAG antibody (clone FG4R) was purchased from Thermo Fisher Scientific and a goat anti-mouse horseradish peroxidase conjugated antibody (clone HAF007) was purchased from R&D systems. Western blotting was done to confirm the ability of the anti- RNA polymerase β-subunit to detect the β-subunit of the RNAP complex in *M. smegmatis* and *M. tuberculosis* (Additional file 1: Figure S6) and to verify the expression of FLAG-tagged proteins (Additional file 1: Figure S5). Oligonucleotides used in this study were purchased from Integrated DNA Technologies and sequences can be found within Additional file 2: Table S9.

### Immunoprecipitation of Nucleoproteins

*M. smegmatis* cultures (2 × 50 mL) grown to an OD_600_ of 0.4 were treated with formaldehyde (1% final concentration) for 10 min at 37 °C with shaking. Cross-linking was quenched using glycine (final concentration 125 mM) and cells were washed using Tris-buffered saline (20 mM Tris-HCl pH 7.5, 150 mM NaCl) prior to storage at − 80 °C. Individual cell pellets were resuspended in 4 mL immunoprecipitation buffer I (IP buffer I) (100 mM Tris-HCl pH 7.5, 300 mM NaCl, 2% Triton X-100) supplemented with protease inhibitors (Roche cOmplete™ mini EDTA-free protease inhibitor cocktail). Cells were subsequently sonicated (QSonica Q700 probe sonicator) four times for 20 s at an amplitude of 30 with 2 min intervals on ice to lyse cells. Mycobacterial DNA was further fragmented through the addition of micrococcal nuclease (100 U, Roche), CaCl_2_ (9 mM) and RNAse A (0.002 mg/mL) followed by incubation at 4 °C for 1 h with rotation. DNA fragmentation was stopped with the addition of EDTA (10 mM) and insoluble cellular debris was removed by centrifugation. Cell lysates were pooled prior to incubation of 2 mL of cell lysate with either 50 μL of Protein G Dynabeads or 5 μg anti-RNAP β-subunit antibody or 10 μg anti-MYH7 antibody immobilized on 50 μL Protein G Dynabeads, respectively. Immunoprecipitations were incubated with an excess cell lysate to fully saturate antibody binding during pull-downs. Nucleoprotein complexes were immunoprecipitated for 2 h at 4 °C, with rotation. Beads were washed twice with IP buffer II (50 mM HEPES-KOH pH 7.5, 150 mM NaCl, 1 mM EDTA, 1% Triton X-100, 0.1% Sodium deoxycholate, 0.1% SDS) supplemented with protease inhibitors, twice with IP buffer II plus 500 mM NaCl, twice with IP buffer II plus 750 mM NaCl, and twice with wash buffer IV (10 mM Tris-HCl pH 8.0, 250 mM LiCl, 1 mM EDTA, 0.5% IGEPAL® CA-630, 0.5% Sodium deoxycholate). Immunoprecipitated nucleoprotein complexes were subjected to on-bead tryptic digestion by incubation of beads with 200 μL 50 mM ammonium bicarbonate and 2 μg sequencing-grade modified trypsin (Promega) for 18 h at 37 °C with shaking at 700 rpm. Eluted peptides were desalted before mass spectrometry analysis using in-house packed STAGE-tips. Samples were concentrated prior to being loaded onto methanol-activated and 2% acetonitrile equilibrated Empore™ Octadecyl C18 STAGE-tips. STAGE-tips were washed with 2% acetonitrile and 0.1% formic acid prior to elution of peptides using a solution of 50% acetonitrile and 0.1% formic acid. Eluted samples were dried using a Concentrator*plus* (Eppendorf) before being resuspended in loading solvent (2% acetonitrile and 0.1% formic acid). Immunoprecipitation experiments were performed in biological triplicate experiments.

### Tandem mass spectrometry analysis

Liquid chromatography was performed using a Dionex-UltiMate 3000 Rapid Separation LC (Thermo Fisher Scientific) equipped with a 2 cm × 100 μm C18 trap column and a custom 35 cm × 75 μm C18 analytical column (Luna C18, 5 μm, Phenomenex). Peptide samples were loaded onto the trap column using 100% Solvent A (2% acetonitrile, 0.1% formic acid) at a flow rate of 5 μl/min using a temperature controlled autosampler set at 7 °C. The trap column was washed for 10 min before elution at 350 nL/min using the following gradient: 2–10% solvent B (99.9% acetonitrile, 0.1% formic acid solution) over 5 min, 10–25% solvent B over 45 min, 25–45% solvent B over 15 min, using Chromeleon™ 6.80 non-linear gradient 6. The column was subsequently washed for 10 min with 80% solvent B solution followed by equilibration using solvent A. Chromatography was performed at 50 °C and the outflow was delivered to the mass spectrometer through a stainless steel nano-bore emitter. Mass spectrometry analysis was performed on the Orbitrap Fusion™ Tribrid™ Mass Spectrometer (Thermo Fisher Scientific) and data was collected in positive mode with a spray voltage set to 2 kV and ion transfer capillary set to 275 °C. Spectra was internally calibrated using polysiloxane ions at m/z = 445.12003 and 371.10024. For MS1 scan analysis, the Orbitrap detector was set to a resolution of R = 120,000 over a scan range of 350–1650 with the AGC target at 3E5 with a maximum injection time of 40 ms. Data was acquired in profile mode. MS2 acquisition was performed using monoisotopic precursor selection for ion charges between + 2 and + 6 with the error tolerance set to +/− 10 ppm and the exclusion of precursor ions from repeat fragmentation for 30 s. Precursor ions were selected for fragmentation using the quadrupole mass analyser and fragmented using an HCD energy of 32.5%. Fragment ions were detected within the ion trap mass analyser using a rapid scan rate. The AGC target was set at 1E4 with a maximum injection time of 45 ms. Data was acquired in centroid mode.

### Identification of immunoprecipitated proteins

MaxQuant 1.5.0.25 was used to analyse mass spectrometry data using the *M. smegmatis* mc^2^ 155 database (UP000000757) containing 8794 predicted protein entries obtained from UniProt, October 2014 [44]. Carbamidomethyl cysteine was set as a fixed modification. Formaldehyde treatment of cells is known to result in the modification of any free nucleophilic group and to minimize the loss of protein identifications due to formaldehyde treatment, possible formaldehyde-induced modifications were searched against LC-MS/MS data, to determine their respective frequencies within our anti-RNAP immunoprecipitation data (Additional file 2: Table S1) [45, 46]. The four most frequent variable modifications (oxidized methionine, the addition of glycine on lysine, serine and threonine residues, the addition of methylol and glycine on any histidine, asparagine, glutamine, tryptophan and tyrosine as well as the possible di-methylation of lysine and arginine residues) were included in our automated database search using MaxQuant. Two missed tryptic cleavages were allowed, and proteins were identified with a minimum of 1 unique peptide detected per protein. The protein and peptide false discovery rate (FDR) set at less than 0.01. Relative quantification was performed using the MaxQuant LFQ (MaxLFQ) algorithm in the MaxQuant package to obtain LFQ intensity values for identified protein groups and the “match between runs” algorithm was selected to detect peptides which were not selected for MS/MS analysis in other experiments [47]. LFQ intensity data for identified proteins from the proteinGroups.txt file was used for statistical analyses using Perseus [47, 48]. All potential contaminants, reverse hits and proteins only identified by site were removed before log 2 transformation and filtering to remove all proteins identified with only one unique peptide [49]. Hierarchical clustering in Perseus was done using the principal component analysis function to demonstrate separate clustering of control immunoprecipitations to anti-RNAP immunoprecipitations for protein groups identified with at least two unique peptides.

Proteins were deemed enriched when present in at least two of the three anti-RNAP immunoprecipitations. Hierarchical clustering of data was performed using Heatmapper (http://heatmapper.ca/) to demonstrate separate clustering of anti-RNAP and control immunoprecipitations and to visually asses the identification of contaminant proteins [50]. A list of high confidence proteins was generated for all proteins identified in two of the three anti-RNAP immunoprecipitations but not in any of the control immunoprecipitations (protein G Dynabead and anti-MYH7 Dynabead controls). Following the removal of high confidence proteins from the dataset, the data was imputed using the “replace missing values from normal distribution” function. A multiple-sample test ANOVA between groups (group 1: anti-RNAP IP, group 2: anti-MYH7 IP, group 3: protein G Dynabead IP) with an FDR of 0.05 was performed using the Benjamini-Hochberg correction. Low confidence proteins were identified as significantly more abundant within the anti-RNAP IPs vs. the control IPs, with a fold-change of at least 2. Identification of specific protein interactions were assessed using CRAPome to identify non-specific protein interactions. CRAPome identified the majority of low confidence and contaminant proteins (Supplementary CRAPome analysis) [51]. All high and low confidence proteins identified with a minimum of 2 unique peptides were subjected to manual spectral inspection. Several proteins were excluded due to poor posterior error probability scores (PEP), major unexplained peaks, poor peptide coverage or low intensity peaks. Identifying characteristics like MS/MS count, number of unique peptides, variable modifications and identifying peptides of all high and low confidence proteins are described in Additional file 2: Table S3 and Table S4.

MSMEG annotations and protein descriptions of identified proteins were assigned using Uniprot (http://www.UniProt.org/) [52]. Gene ontology enrichment analysis was done using the Gene Ontology Enrichment Analysis Software Toolkit (GOEAST) (http://omicslab.genetics.ac.cn/GOEAST/) followed by removal of redundant GO identifications using Reduce and Visualize Gene Ontology (REVIGO) (http://revigo.irb.hr/) [26, 27]. Functional annotation network graphs generated by REVIGO was visualized using Cytoscape 3.3.0 [28] and to demonstrate the enrichment of GO terms associated with nucleic acids we generated graphs by searching for the identifiers DNA, chromosome, transcription, gene, RNA, ribosome, translation, nucleotide as well as their first connecting neighbours.

### Creation of FLAG-tagged protein plasmids

A FLAG-tag and 6x glycine linker was synthesized as part of primer NFLAG0615 f, which was used to PCR amplify MSMEG_0615 together with primer NFLAG0615 r (Additional file 2: Table S9). The resulting fragment was cloned into a modified pSE100 backbone plasmid, which contained a ribosome binding sequence, to create pNFLAG0615. To generate a FLAG-tag only containing plasmid, the insert MSMEG_0615 was excised using NdeI and HindIII, prior to blunting with the Klenow fragment. *M. smegmatis* genes of interest, MSMEG_1060, MSMEG_2695, MSMEG_3754, MSMEG_4306 and MSMEG_5512 were PCR amplified using primers described in Additional file 2: Table S9. Genes of interest were cloned into the linearized pNFLAG0615 plasmid using the In-Fusion® HD Cloning kit. Gene sequence integrity was verified using Sanger sequencing, performed on an ABI 3730XL DNA Analyser at the Central Analytical Facilities, Stellenbosch University, South Africa. pNFLAG plasmids were co-transformed with pTEK-4S-0X into *M. smegmatis*. All plasmids used or generated in this study are described in Additional file 2: Table S10.

### DNA association assay

Expression of N-terminally FLAG-tagged proteins were confirmed by western blotting. Cell lysates were collected from *M. smegmatis* mc^2^155 and *M. smegmatis* pNFLAG, pNFLAG0615, pNFLAG2695, pNFLAG3754, pNFLAG4306 and pNFLAG5512 transformants. Samples were separated on 4–12% gradient SDS-PAGE gels before being transferred to a PVDF membrane and probed using an anti-FLAG antibody (1:4000) and a goat anti-mouse secondary antibody (1:10000).

Overnight cultured *M. smegmatis* strains expressing the FLAG-tagged proteins were cross-linked and lysed as described above. The FLAG-tagged proteins were immunoprecipitated from cell lysates using 5 μg of mouse anti-FLAG primary antibody (clone FG4R) immobilized on protein G Dynabeads™. Antibody-bound beads were incubated with 2 mL cell lysate for 2 h at 4 °C before being washed as previously described. Cross-linking was reversed by incubating beads in 100 μL elution buffer (50 mM Tris-HCl pH 7.5, 10 mM EDTA, 1% SDS) for 1 h at 65 °C. Proteins were digested with proteinase K prior to NaCl - ethanol DNA precipitation. DNA was resuspended in 20 μL TE buffer (10 mM Tris-HCl pH 7.5, 1 mM EDTA) prior to separation on an agarose gel.

## Supplementary information

**Additional file 1: Figure S1.** Hierarchical clustering of enriched biological processes GO terms. Hierarchical clustering of GO terms associated with biological processes showed enrichment for GO terms associated with nucleic acids such as DNA replication, DNA metabolic process, gene expression and translation. Higher hierarchical GO terms are displayed in black and lower hierarchical GO terms in white. **Figure S2.** Hierarchical clustering of enriched molecular function GO terms. Hierarchical clustering of GO terms associated with molecular functions showed enrichment for GO terms associated with nucleic acids, including nucleotide binding, nucleic acid binding, RNA binding and DNA-dependent ATPase activity. Higher hierarchical GO identities are displayed in black with lower hierarchical GO identities displayed in white. **Figure S3.** Hierarchical clustering of enriched cellular component GO terms. Hierarchical clustering of GO terms associated with cellular components demonstrated an enrichment for ribosomal GO terms. Higher hierarchical GO terms are displayed in black and lower hierarchical GO terms in white. **Figure S4.** Metabolic pathway mapping of AP-MS identified proteins. AP-MS identified proteins were mapped using KEGG metabolic pathway mapping. Identified proteins were shown to be present in metabolic pathways associated with energy, lipid, carbohydrate, amino acid, and nucleotide metabolism. Enriched pathways are displayed in black. **Figure S5.** Detection of N-terminally FLAG-tagged proteins in *M. smegmatis*. Western blotting was used to confirm the expression of FLAG-tagged *M. smegmatis* proteins using an anti-FLAG antibody. Full length FLAG-MSMEG_0615, FLAG-MSMEG_2695, FLAG-MSMEG_3754, FLAG-MSMEG_4306 and FLAG-MSMEG_5512 was detected. HupB is known to form a homodimer and FLAG-MSMEG_2389 could be located at ~ 35 kDa instead of at 22.7 kDa. Likewise FLAG-MSMEG_1060, which shares a high level of sequence similarity with Lsr2 and is also known to form a homodimer, could be identified at ~ 25 kDa and not at 15.83 kDa. **Figure S6.** Detection of RNA polymerase β-subunit in *M. smegmatis* and *M. tuberculosis*. Western blotting was used to confirm the ability of the antibody raised against the *E. coli* RNA polymerase β-subunit to detect this subunit in *M. smegmatis* (128.53 kDa) and *M. tuberculosis* (129.21 kDa)*.* The ability of this antibody to recognise the β-subunit of the RNAP complex in *M. smegmatis* was also confirmed with mass spectrometry (Additional file 2: Table S2).

**Additional file 2: Table S1.** Prevelance of formaldehyde crosslinking and glycine quencing variable modifications. **Table S2.** High and low confidence proteins. **Table S3.** Identifying characteristics of high and low confidence proteins. **Table S4.** Unique peptides of identified high and low confidence proteins. **Table S5.** Potential low confidence proteins. **Table S6.** Gene ontology enrichment of non-redundant biological processes GO terms. **Table S7.** Gene ontology enrichment of non-redundant molecular function GO terms. **Table S8.** Gene ontology enrichment of non-redundant cellular component GO terms. **Table S9.** Cloning and sequencing primers. **Table S10.** Plasmids.

## Data Availability

The mass spectrometry proteomics data have been deposited to the ProteomeXchange Consortium via the PRIDE partner repository with the dataset identifier PXD016241 [53]. Analysed data from this study are included in this published article and its supplementary information files.

## Abbreviations

AD: Albumin Dextrose
ANOVA: Analysis of Variance
AP-MS: Affinity Purification-Mass Spectrometry
ChIP-seq: Chromatin Immunoprecipitation Sequencing
DNA: Deoxyribonucleic Acid
*E. coli*: *Escherichia coli*
eRIC: Enhanced RNA Interactome Capture
FDR: False Discovery Rate
GO: Gene Ontology
GOEAST: Gene Ontology Enrichment Analysis Software Toolkit
IP: Immunoprecipitation
LB: Luria-Bertani
LFQ: Label-free quantification
*M. smegmatis*: *Mycobacterium smegmatis*
MYH7: Human Myosin Heavy Chain 7
OOPS: Orthogonal Organic Phase Separation
PEP: Posterior Error Probability Scores
PVDF: Polyvinylidene Fluoride
REVIGO: Reduce Visualize Gene Ontology
RNA: Ribonucleic Acid
RNA-seq: RNA sequencing
RNAP: RNA polymerase complex
USA: United States of America
