# Supplementary material for: Identifying nucleic acid-associated proteins in Mycobacterium smegmatis by mass spectrometry-based proteomics
Source: BMC Mol Cell Biol. 2020 Mar 23;21:19. doi: 10.1186/s12860-020-00261-6 (PMC7092591; doi:10.1186/s12860-020-00261-6)
Supplement: Supplementary file 1 — Additional file 1: Figure S1. Hierarchical clustering of enriched biological processes GO terms. Hierarchical clustering of GO terms associated with biological processes showed enrichment for GO terms associated with nucleic acids such as DNA replication, DNA metabolic process, gene expression and translation. Higher hierarchical GO terms are displayed in black and lower hierarchical GO terms in white. Figure S2. Hierarchical clustering of enriched molecular function GO terms. Hierarchical clustering of GO terms associated with molecular functions showed enrichment for GO terms associated with nucleic acids, including nucleotide binding, nucleic acid binding, RNA binding and DNA-dependent ATPase activity. Higher hierarchical GO identities are displayed in black with lower hierarchical GO identities displayed in white. Figure S3. Hierarchical clustering of enriched cellular component GO terms. Hierarchical clustering of GO terms associated with cellular components demonstrated an enrichment for ribosomal GO terms. Higher hierarchical GO terms are displayed in black and lower hierarchical GO terms in white. Figure S4. Metabolic pathway mapping of AP-MS identified proteins. AP-MS identified proteins were mapped using KEGG metabolic pathway mapping. Identified proteins were shown to be present in metabolic pathways associated with energy, lipid, carbohydrate, amino acid, and nucleotide metabolism. Enriched pathways are displayed in black. Figure S5. Detection of N-terminally FLAG-tagged proteins in M. smegmatis. Western blotting was used to confirm the expression of FLAG-tagged M. smegmatis proteins using an anti-FLAG antibody. Full length FLAG-MSMEG_0615, FLAG-MSMEG_2695, FLAG-MSMEG_3754, FLAG-MSMEG_4306 and FLAG-MSMEG_5512 was detected. HupB is known to form a homodimer and FLAG-MSMEG_2389 could be located at ~ 35 kDa instead of at 22.7 kDa. Likewise FLAG-MSMEG_1060, which shares a high level of sequence similarity with Lsr2 and is also known to form a homodimer, coul [file 12860_2020_261_MOESM1_ESM.pdf]

Supplementary Information for:

Identifying nucleic acid-associated proteins in Mycobacterium smegmatis by mass spectrometry-based proteomics

Nastassja L. Kriel1*, Tiaan Heunis1,2, Samantha L. Sampson1, Nico C. Gey van Pittius1, Monique J. Williams1**, Robin M. Warren1**

[1] DST-NRF Centre of Excellence for Biomedical Tuberculosis Research; South African Medical Research Council Centre for Tuberculosis Research; Division of Molecular Biology and Human Genetics, Faculty of Medicine and Health Sciences, Stellenbosch University, Cape Town.
[2] Institute for Cell and Molecular Biosciences, Newcastle University, Newcastle upon Tyne, United Kingdom.

*Corresponding author. Mailing address: Division of Molecular Biology and Human Genetics, Faculty of Medicine and Health Sciences, Stellenbosch University. PO Box 19063 Tygerberg, 7505 Cape Town, South Africa. Phone: +(27)-21-9389073 Fax: +(27)-21-9389476. Email: nastassja@sun.ac.za
**Co-senior authors.

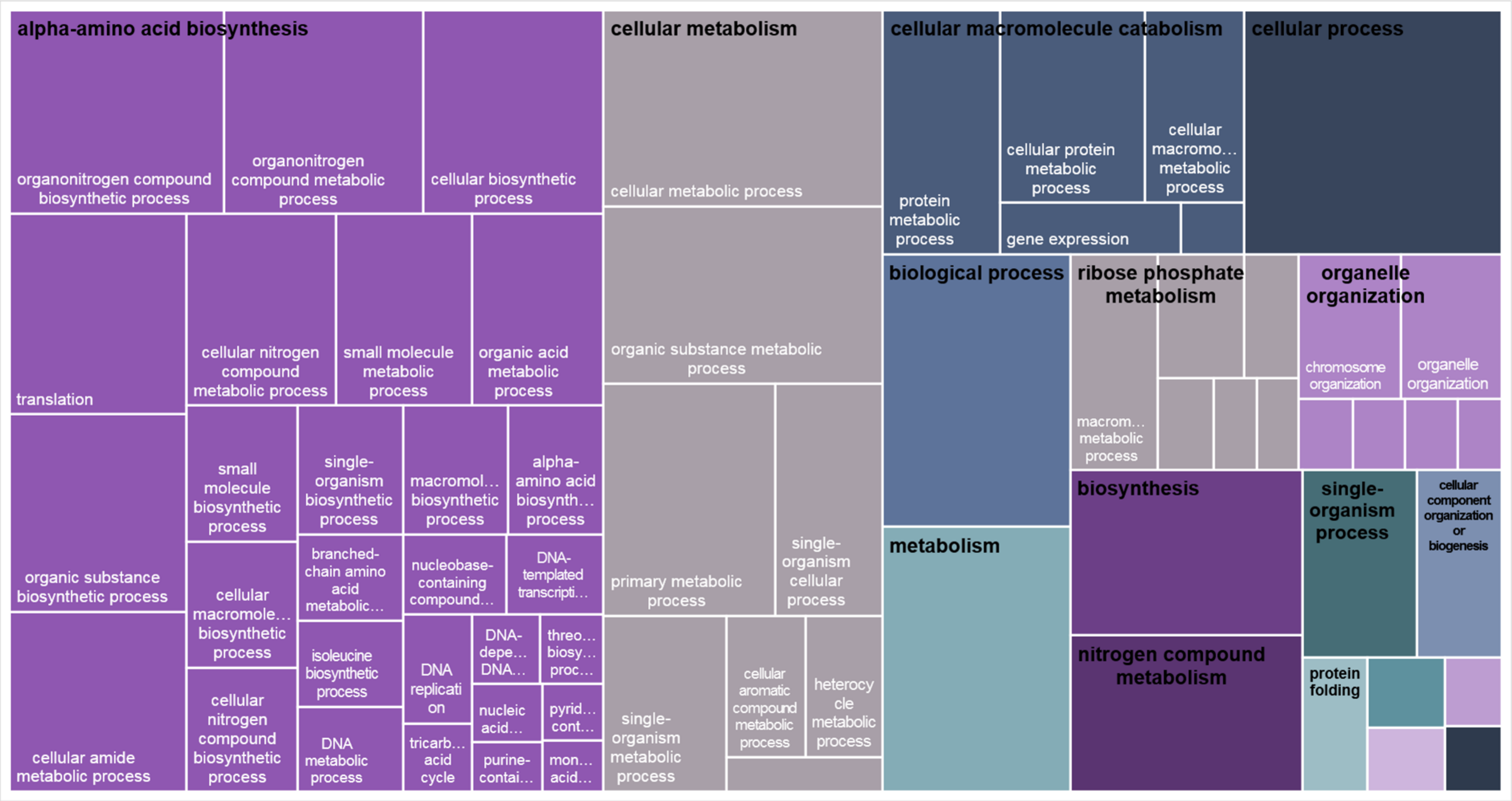

Figure S1. Hierarchical clustering of enriched biological processes GO terms. Hierarchical clustering of GO terms associated with biological processes showed enrichment for GO terms associated with nucleic acids such as DNA replication, DNA metabolic process, gene expression and translation. Higher hierarchical GO terms are displayed in black and lower hierarchical GO terms in white.

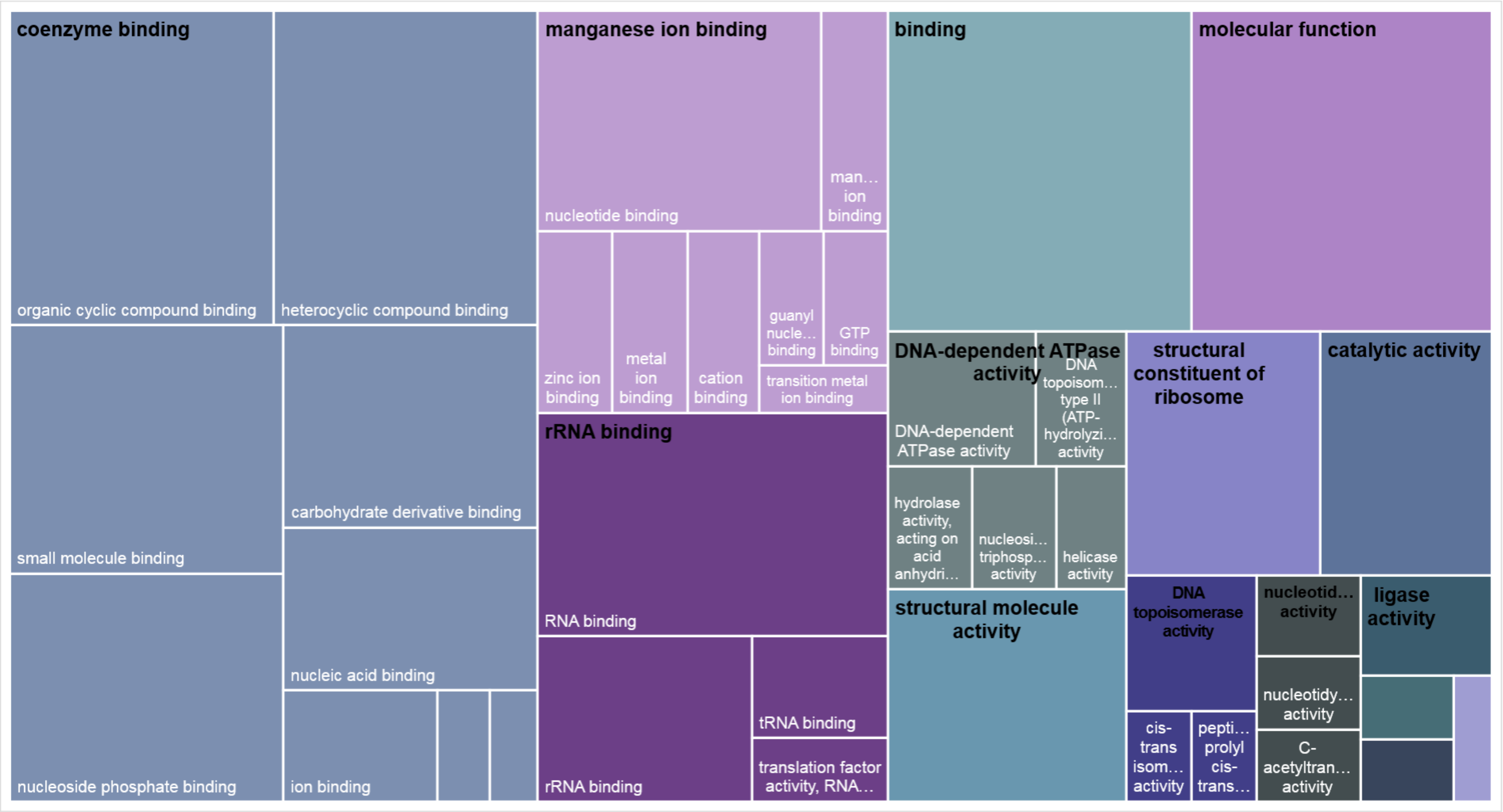

Figure S2. Hierarchical clustering of enriched molecular function GO identities terms. Hierarchical clustering of GO terms associated with molecular functions showed enrichment for GO terms associated with nucleic acids, including nucleotide binding, nucleic acid binding, RNA binding and DNA-dependent ATPase activity. Higher hierarchical GO identities are displayed in black with lower hierarchical GO identities displayed in white.

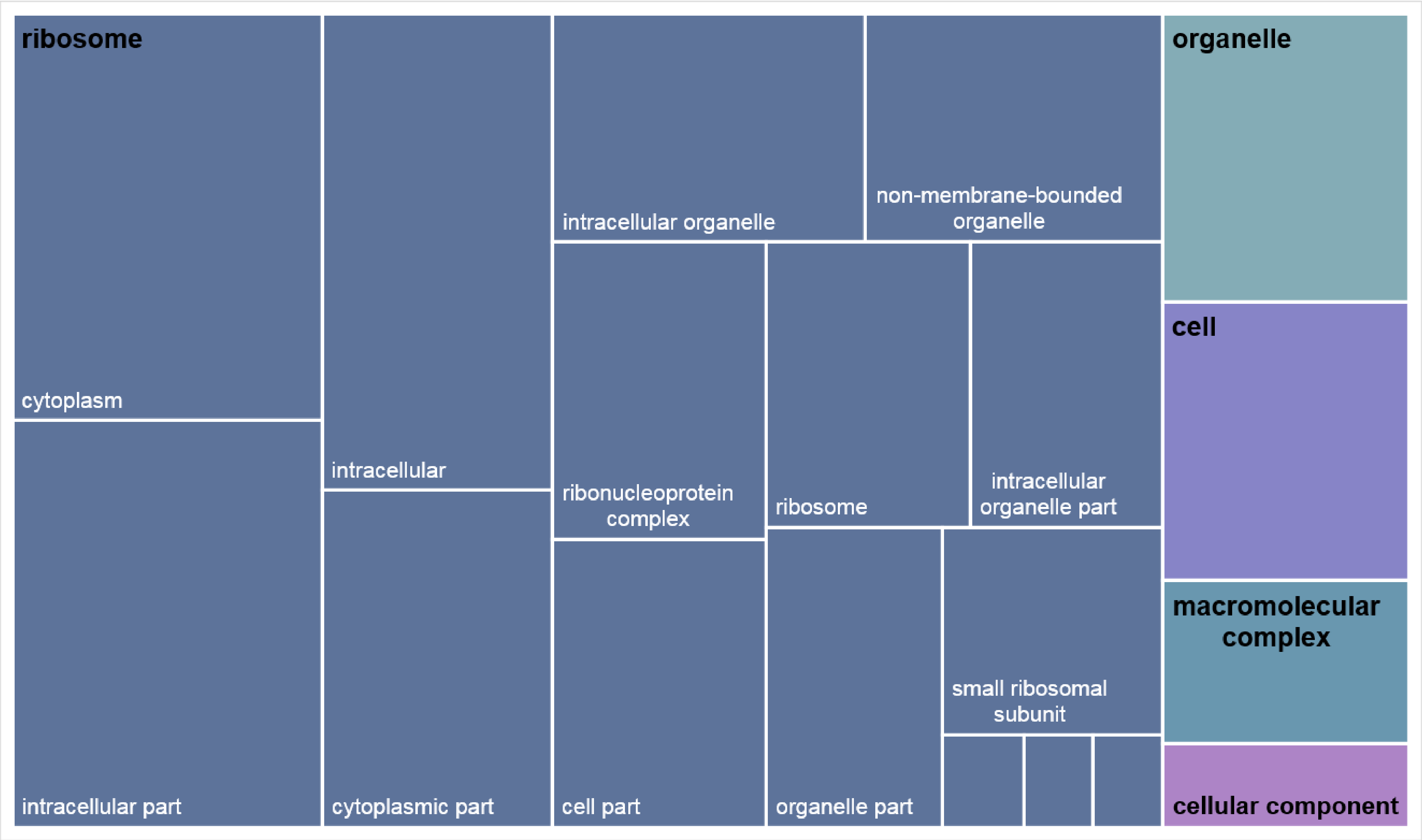

Figure S3. Hierarchical clustering of enriched cellular component GO terms. Hierarchical clustering of GO terms associated with cellular components demonstrated an enrichment for ribosomal GO terms. Higher hierarchical GO terms are displayed in black and lower hierarchical GO terms in white.

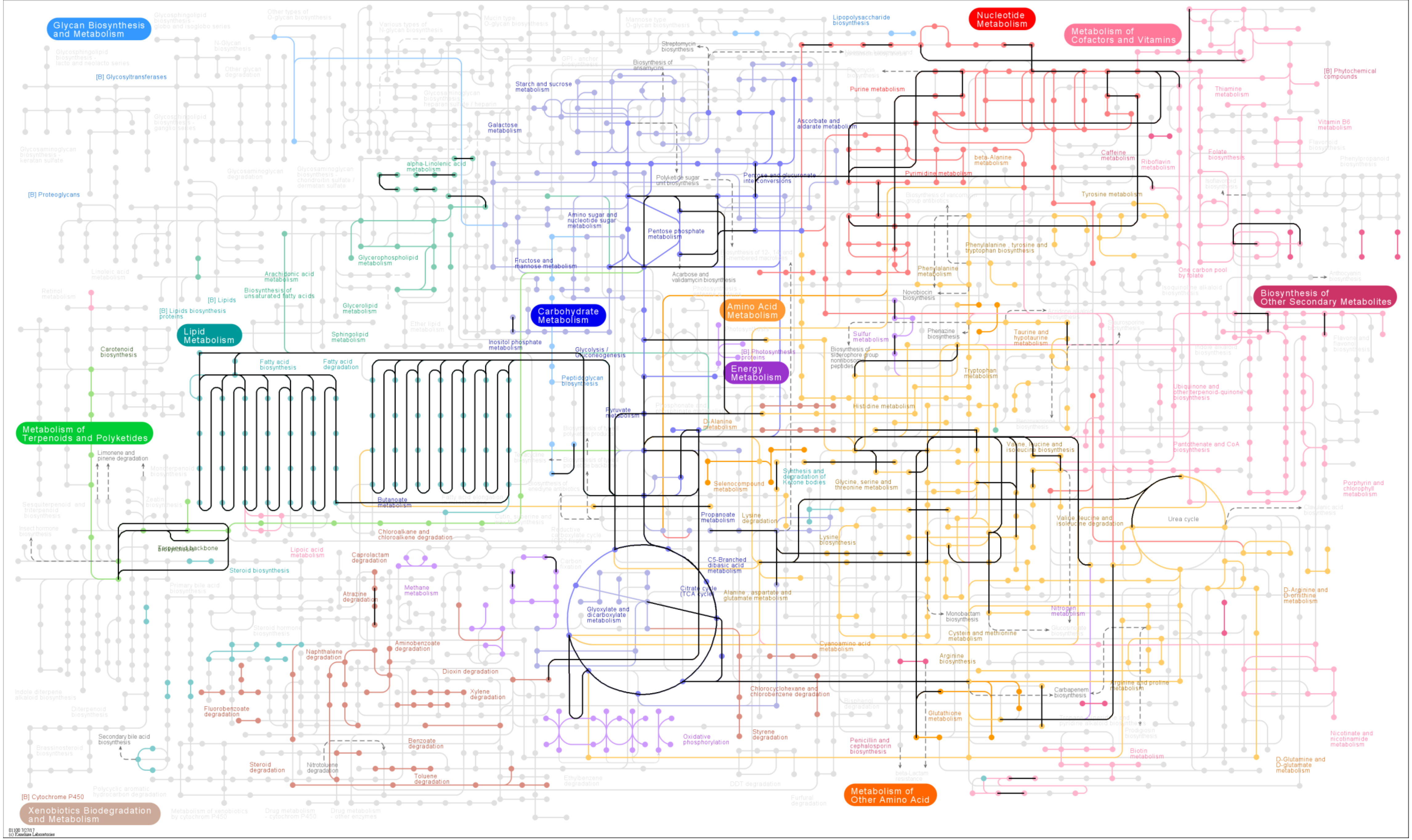

Figure S4. Metabolic pathway mapping of AP-MS identified proteins. AP-MS identified proteins were mapped using KEGG metabolic pathway mapping. Identified proteins were shown to be present in metabolic pathways associated with energy, lipid, carbohydrate, amino acid, and nucleotide metabolism. Enriched pathways are displayed in black.

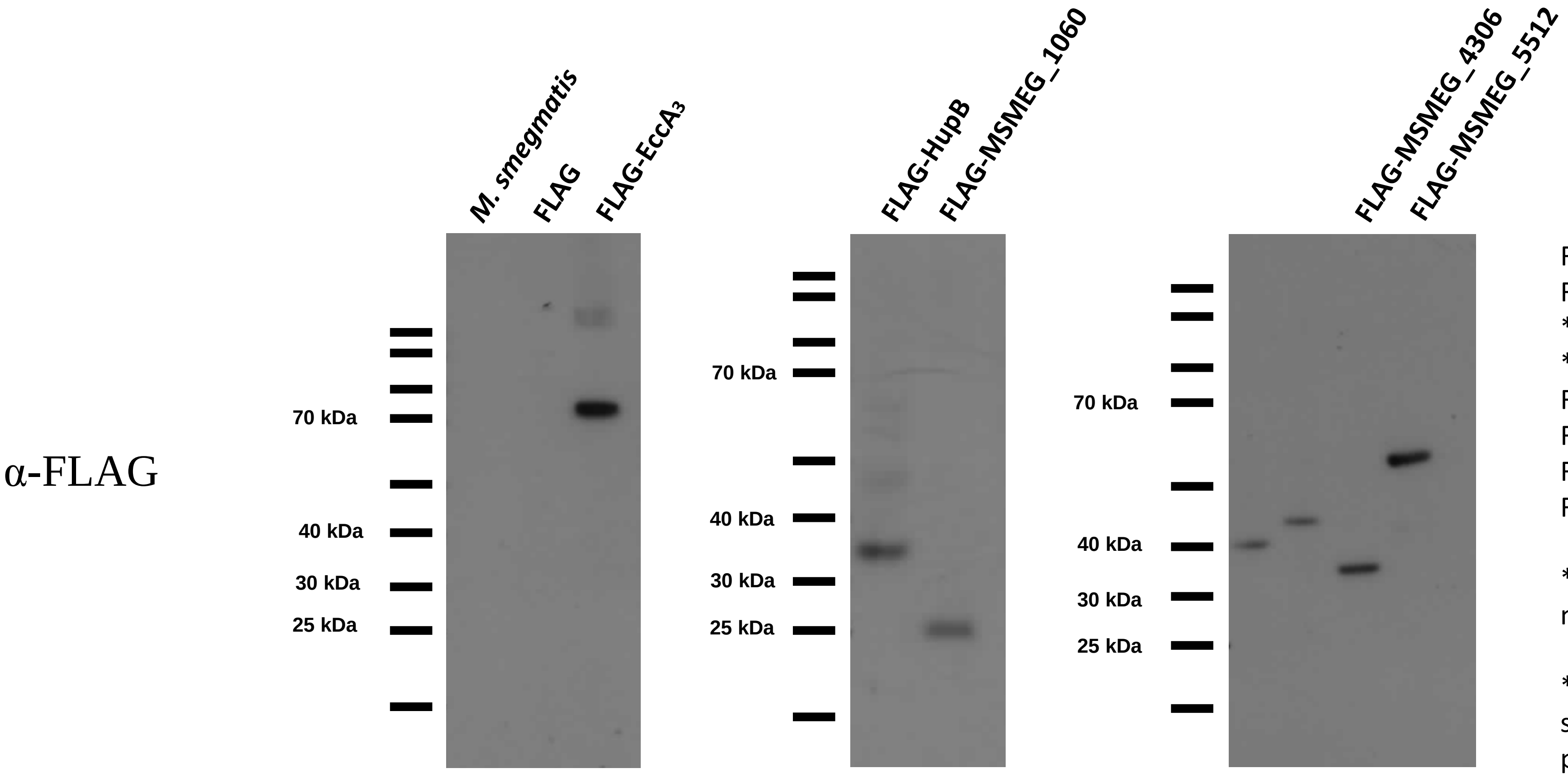

FLAG-tag	1.5 kDa
FLAG-EccA3	69.83 kDa
*FLAG-HupB	22.7 kDa
**FLAG-MSMEG_1060	15.83 kDa
FLAG-MSMEG_2695	31.8 kDa
FLAG-MSMEG_3754	37.04 kDa
FLAG-MSMEG_4306	28.61 kDa
FLAG-MSMEG_5512	51.5 kDa

*HupB is known to form a dimer which may not have resolved.

** MSMEG_1060 shares high sequence similarity with the nucleoid associated protein Lsr2, which is known to form a dimer. Dimer may not have resolved.

Figure S5. Detection of N-terminally FLAG-tagged proteins in *M. smegmatis*. Western blotting was used to confirm the expression of FLAG-tagged *M. smegmatis* proteins using an anti-FLAG antibody. Full length FLAG-MSMEG_0615, FLAG-MSMEG_2695, FLAG-MSMEG_3754, FLAG-MSMEG_4306 and FLAG-MSMEG_5512 was detected. HupB is known to form a homodimer and FLAG-MSMEG_2389 could be located at ~35 kDa instead of at 22.7 kDa. Likewise FLAG-MSMEG_1060, which shares a high level of sequence similarity with Lsr2 and is also known to form n a homodimer, could be identified at ~25 kDa and not at 15.83 kDa.

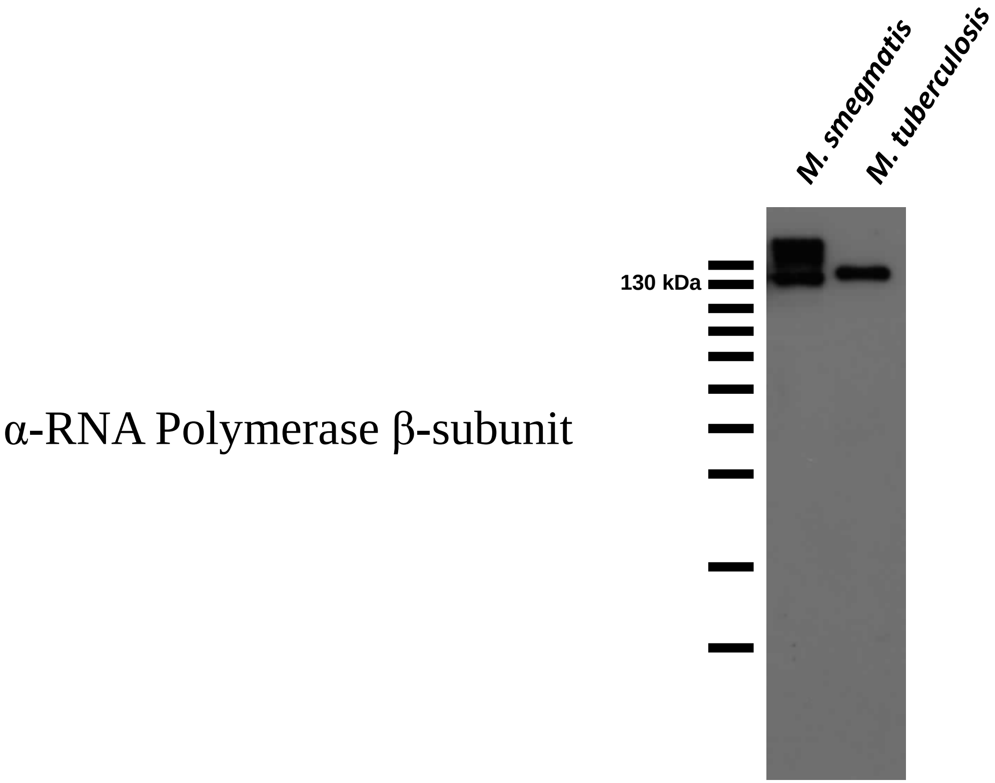

Figure S6. Detection of RNA polymerase β-subunit in *M. smegmatis* and *M. tuberculosis*. Western blotting was used to confirm the ability of the antibody raised against the *E. coli* RNA polymerase β-subunit to detect this subunit in *M. smegmatis* (128.53 kDa) and *M. tuberculosis* (129.21 kDa). The ability of this antibody to recognise the β-subunit of the RNAP complex in *M. smegmatis* was also confirmed with mass spectrometry (Table S2).
